# Association between phase angle and the nutritional status in pediatric populations: a systematic review

**DOI:** 10.3389/fnut.2023.1142545

**Published:** 2023-06-21

**Authors:** Andrea Franco-Oliva, Azalia Ávila-Nava, Estíbaliz Amairani Rodríguez-Aguilar, Ander Trujillo-Mercado, Alda Daniela García-Guzmán, Beatriz Adriana Pinzón-Navarro, Jimena Fuentes-Servín, Martha Guevara-Cruz, Isabel Medina-Vera

**Affiliations:** ^1^Departamento de Metodología de la Investigación, Instituto Nacional de Pediatría, Ciudad de México, Mexico; ^2^Hospital Regional de Alta Especialidad de la Península de Yucatán, Mérida, Mexico; ^3^Tecnologico de Monterrey, Escuela de Medicina y Ciencias de la Salud, Ciudad de México, Mexico; ^4^Servicio de Oncología Médica, Instituto Nacional de Pediatría, Ciudad de México, Mexico; ^5^Servicio de Gastroenterología y Nutrición Pediátrica, Instituto Nacional de Pediatría, Ciudad de México, Mexico; ^6^Departamento de Fisiología de la Nutrición, Instituto Nacional de Nutrición y Ciencias Médicas Salvador Zubirán, Ciudad de México, Mexico

**Keywords:** phase angle, children, adolescent and youth, nutritional status, pediatric population

## Abstract

**Background and aims:**

Malnutrition is prevalent in pediatric populations with any disease, and it is also related to changes in body composition. In addition, recent studies have documented relationships between these changes and phase angle (PhA), an important parameter of functional nutritional assessment. PhA could be a new marker of nutritional status. Many studies have generated information about the association between PhA and malnutrition in various pathologies, although the vast majority of this information is from adult populations. In this systematic review, we answered the following question: What is the association between PhA and the nutritional status in pediatric populations?

**Methods:**

We performed a systematic search of the Medline/PubMed and Latin American and Caribbean Health Sciences Literature databases (LILACS) databases for studies published up to October 2022. The inclusion criteria were pediatric subjects, which reported the relationship between PhA and the nutritional status with any objective nutritional indicator, and PhA was measured by electric impedance and reported at 50 kHz. We synthesized data from the studies that reported cutoff analysis of PhA with receiver operating characteristic (ROC) curves, mean PhA values presented by nutritional status strata, and correlations between PhA and nutritional status indicators. We assessed the risk of bias by using the Quality Assessment Tool for Observational Cohort and Cross-Sectional Studies and the Quality Assessment for Diagnostic Accuracy Studies.

**Results:**

Of the 126 studies we identified, 15 met the inclusion criteria. The included studies reported the association between PhA and objective indicators of nutritional status, including weight-for-age z-score (WAZ) <-1 standard deviation (SD) for malnutrition, height-for-age z-score (HAZ) for malnutrition-stunting, body mass index (BMI) for the starvation state, body mass index z-score (BMIz) and BMI for malnutrition, mid-upper arm circumference (MUAC) <11 cm for severe acute malnutrition (SAM), and fat-free mass index z-score (FFMIz) <-2 z-score for moderate malnutrition, among others. The report of these associations between PhA and nutritional status was based on cutoff points generated with ROC curve analysis or comparison of mean PhA values, which were reported stratified by the presence or absence of malnutrition, and correlations between PhA and anthropometric indicators for the evaluation of the nutritional status in the pediatric population. It was difficult to compare the studies due to the heterogeneity of the bioelectrical impedance analysis models used, how PhA was reported (standardized, percentiles, or degrees), and the anthropometric indicators used to diagnose malnutrition.

**Conclusion:**

The early identification of malnutrition is relevant to establish the correct nutritional treatment; PhA appears to be a sensitive indicator of nutritional status and is easy to obtain. Although the results of this review are inadequate to establish PhA cutoff points associated with malnutrition in pediatric populations, in most of the studies, there was an association between PhA and objective indicators of nutritional status.

**Systematic review registration:**

https://www.crd.york.ac.uk/prospero/display_record.php?ID=CRD42022362413, identifier: PROSPERO 2022 CRD42022362413.

## Introduction

Malnutrition is prevalent in pediatric populations with any disease. It affects normal growth and response to treatment, as well as other clinical outcomes. Considering the irreversible damage of malnutrition in the pediatric stage, it is essential to assess the nutritional status and ensure an adequate nutritional supply to manage a disease. The nutritional status in pediatric patients has generally focused on evaluation based on anthropometric parameters such as body weight (BW) and height: Individual values are compared with standard growth curves of the population (1). Other anthropometric indicators used to assess the nutritional status are weight-for-age which evaluates wasting (acute malnutrition), height-for-age which evaluates stunting (chronic malnutrition), and body mass index (BMI) compared with appropriate growth charts (percentiles or z-scores). Mid-upper arm circumference (MUAC) and triceps skin fold (TSF) have also been used, but they require a trained professional to obtain the measurements. However, to date, there is not a uniform definition of malnutrition in children. The interdisciplinary American Society for Parenteral and Enteral Nutrition (ASPEN) Working Group defined pediatric malnutrition (undernutrition) as an imbalance between nutrient requirements and intake, resulting in cumulative deficits of energy, protein, or micronutrients that may negatively affect growth, development, and other relevant outcomes (2). Even with this work, there is no consensus as to which anthropometric parameters are ideal. Nevertheless, there are other strategies related to the nutritional status that are based on body composition such as bioelectrical impedance analysis (BIA). This indirect, non-invasive, and easy-to-apply technique is used to measure body compartments such as body cell mass (BCM), fat-free mass (FFM), fat mass (FM), and total body water (TBW). BIA measurements are based on the transition of electrical current through the body to estimate the TBW, which, in turn, is based on the hydration constants of the tissues. FFM is obtained and FM is derived by using a simple equation based on two components (FFM [kg] = total weight [kg]—FM [kg]) (3).

Recent studies have documented changes in the relationship between body composition and phase angle (PhA), an important parameter of functional nutritional assessment. PhA is also measured by BIA, but it is calculated based on the relationship between the indicators of the crude electrical parameters including impedance (Z), resistance (R), and reactance (Xc): PhA = {[arc tangent (Xc/R)] × 180°/π} (4). R measures the opposition of the cell to the passage of electric current; it is determined by the state of hydration of the cell and tissue. The higher the hydration, the lower the R. Xc measures the electrical charge of the system or the capacity of the cell to store energy; it is determined by the cell membrane and the cell size. The greater the integrity of the membrane and greater the cellularity, the higher the Xc (5). PhA can be negatively influenced by various clinical diseases such as malnutrition. Because PhA is a marker of the quantity and quality of soft tissue mass, as well as the hydration status, many authors consider it a useful marker of nutritional status. In disease-related malnutrition, the characteristic early shift from intracellular water (ICW) to extracellular water (ECW) and an increased ratio of extracellular to BCM are reflected in PhA (6). Alteration of the electrical properties of the tissue that are detectable with BIA has been associated with the presence of malnutrition related to disease (7).

Based on these findings, PhA could identify malnutrition early due to its sensitivity to detect changes in body composition with respect to anthropometric measurements (8). Many studies have generated PhA cutoff points to identify malnutrition in various pathologies, although the vast majority of these studies have been in adults (9–11). PhA cutoff points have also been generated but are associated with survival indicators as a reference standard (12–14). Other researchers have shown how PhA is associated with various anthropometric indicators in pediatric populations, but they have not provided cutoff points (15, 16). The association between PhA and nutritional status is highly variable due to the lack of uniform definitions for malnutrition and because various anthropometric indicators are used for it (2). Hence, in this systematic review, we summarized the evidence regarding the association between PhA and the nutritional status in pediatric populations.

## Methods

We performed a systematic review to answer the following research question: What is the association between PhA and the nutritional status in pediatric populations? We followed the Preferred Reporting Items for Systematic Reviews and Meta-Analysis (PRISMA) guidelines (17). We registered the protocol at the International Prospective Register of Systematic Reviews (PROSPERO) under registration number CRD42022362413. The protocol was approved by the Instituto Nacional de Pediatría Research and Ethics Committees (number 2022/065) and officially registered at the Office for Human Research Protections of the NIH (http://ohrp.cit.nih.gov/search/search.aspx) with numbers IRB00013674 and IRB00013675.

### Search strategy

Two authors (AFO and EARA) performed the search independently. They searched the online MEDLINE/PubMed and Latin American and Caribbean Health Sciences Literature (LILACS) databases. They used the following Medical Subject Headings (MeSH) and other terms related to the subject as part of the search strategy: (“Phase Angle”) AND (Children OR Adolescents OR Pediatrics) AND (Malnutrition OR Undernutrition OR Malnourishment OR “Nutritional Status” OR “Nutrition Status”). They applied no restrictions to the date of publication of papers. They searched for articles published up to October 2022. Table 1 shows the description of the Population, Exposition, Comparators, and Outcomes (PECO) strategy applied in the present systematic review. It is based on pediatric populations (Population); PhA at 50 kHz (Exposition); any objective method to evaluate nutritional status (Comparator); and PhA cutoff points associated with malnutrition (area under the curve [AUC] values, sensitivity, and specificity), comparison of median PhA values among malnutrition strata, and correlations between PhA values and malnutrition indicator values (Outcomes).

**Table 1:** PECO criteria for study selection.

**Criterion**		**Description**
**P**	Population	Children <18 years old
**E**	Exposition	Phase angle at 50 kHz
**C**	Comparator	Objective methods to assess the nutritional status. The comparator was made with objective indicators to assess the state of nutrition, including weight-for-age, height-for-age, weight-for-height, body mass index, mid-upper arm circumference, triceps skinfold thickness, and fat-free mass index.
**O**	Outcomes	•Phase angle cutoff points analyzed with receiver operating characteristic curves associated with malnutrition (area under the curve values, sensitivity, and specificity). •Comparison of median phase angle values among malnutrition strata. •Correlation between phase angle values and malnutrition indicators values.

### Selection of studies

After removing duplicates, the same authors (AFO and EARA) independently screened the titles and abstracts for eligibility evaluation, based on the inclusion criteria. They also carried out data extraction.

### Selection criteria

We included original studies if they met the following criteria: [1] performed on pediatric subjects <18 years of age, [2] reported the relationship between PhA and the nutritional status based on any objective nutritional indicator, [3] measured and reported PhA at 50 kHz, [4] articles written in English or Spanish, and [5] population with any clinical health condition, even a healthy population. The exclusion criteria were: [1] data with adult populations, [2] review articles, and [3] studies reporting PhA reference value tables. We also excluded studies if they contained overlapping subjects with other studies.

### Data extraction and synthesis methods

AFO and EARA independently extracted the following data from each study: [1] first author's name; [2] year of publication; [3] study location; [4] study design; [5] sample size; [6] population sex; [7] population age; [8] clinical health condition; [9] nutritional status indicator; [10] reference for malnutrition; [11] principal results related to the association between PhA and the nutritional status or any nutritional indicator; [12] prevalence of malnutrition with any nutritional indicator; [13] BIA model; [14] BIA usage specifications; [15] PhA estimation formula used; and [16] measurement position reported. A discrepancy between AFO and EARA was resolved by the opinion of another researcher (IMV or AAN). We synthesized the data from the studies that [1] reported the cutoff analysis of PhA with receiver operating characteristic (ROC) curve analysis (reporting the cutoff point associated with poor nutrition, as well as sensitivity and specificity if included), [2] compared mean PhA values between nutritional status strata, and [3] reported correlations between PhA values and nutritional status indicators. It was difficult to make comparisons among the included studies due to the heterogeneity of the data (age ranges, different diseases, and different objective indicators of nutritional status).

### Risk of bias assessment

We assessed the risk of bias in the cohort and cross-sectional studies by using the Quality Assessment Tool for Observational Cohort and Cross-Sectional Studies developed jointly by methodologists from the National Heart, Lung, and Blood Institute (NHLBI) and Research Triangle Institute International (18). The tool assesses potential flaws in the study methodology, including the following sources of bias: patient selection, performance, attrition and detection, confounding, study power, and other factors. A judgment of “good” indicates a low risk of bias, “fair” indicates that the study was susceptible to some bias considered not sufficient to invalidate its results, and “poor” indicates a significant risk of bias. We applied the Quality Assessment of Diagnostic Accuracy Studies (QUADAS-2) (19) to assess the studies that performed concurrent validity analyses. The tool comprises four main domains: patient selection, index test, reference standard, and flow and timing. These domains classify the risk of bias and applicability. The results can be expressed as high, not clear, or low risk of bias. Because there is no reference standard to evaluate the nutritional status in the pediatric population, we considered the anthropometric objective indicators as reference standards.

## Results

### Identification of studies

**Figure 1:**
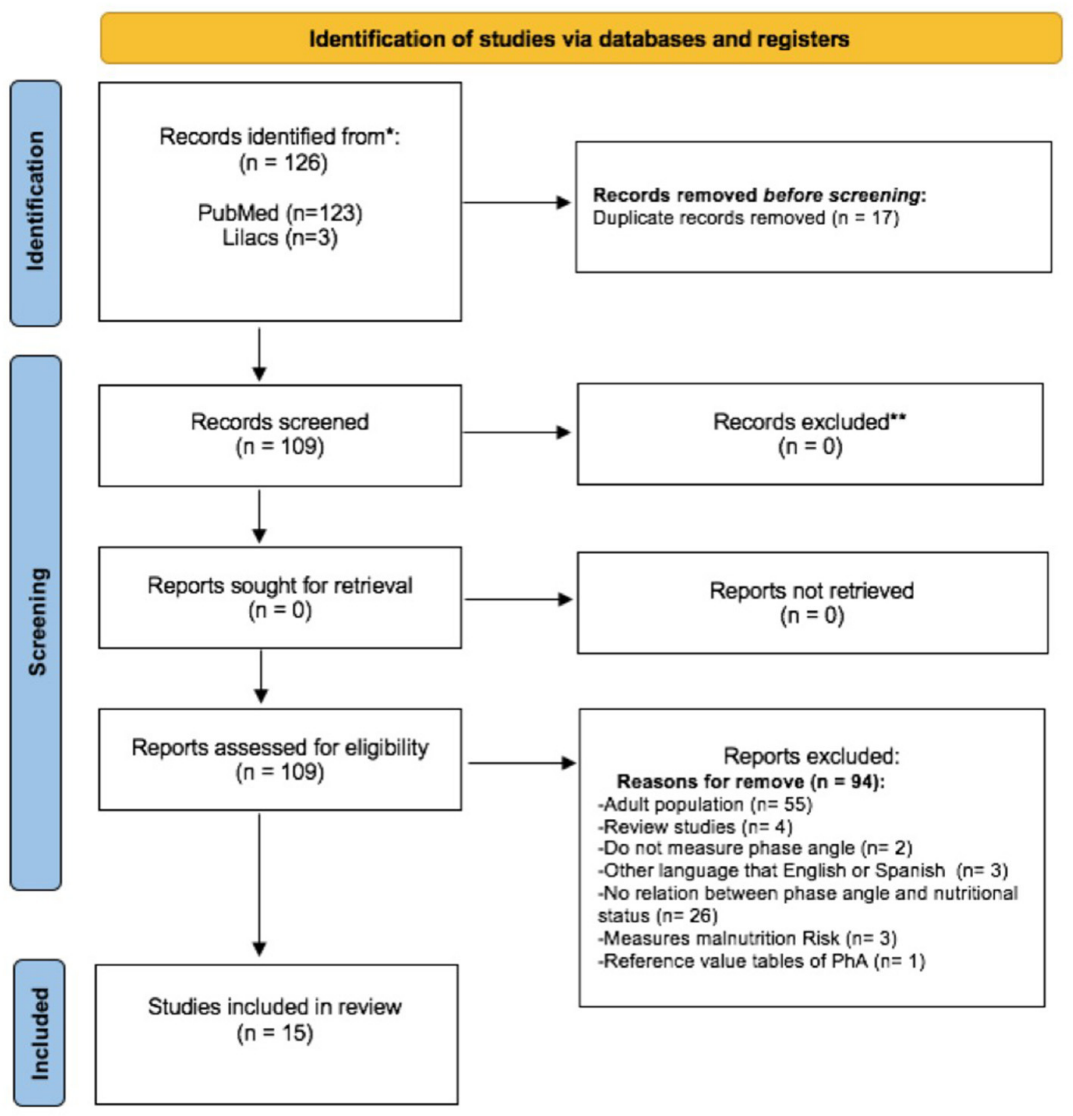
Study selection flowchart (17).

Our search identified 126 possible studies; of these, we excluded 17 duplicates. Of the remaining 109 studies, we excluded 94 that did not meet the inclusion criteria. Hence, we included 15 articles in this review (Figure 1). Five studies were carried out in Brazil; three in the United Kingdom; two in Ethiopia; and one each in Greece, Poland, Italy, Malawi, and Japan. They had been published between 2000 and 2021. All selected studies evaluated male and female subjects, except for one: Popiolek et al. (20), which included only female subjects with anorexia nervosa.

### Description of the studies

The clinical health conditions of patients included in the studies were: five studies evaluated children with malnutrition; two evaluated excess weight in 8-year-old children and indigenous children and adolescents; and the remaining studies concerned hematopoietic stem cell transplantation, chronic kidney disease (CKD), anorexia nervosa, congenital heart disease (CHD), autism spectrum disorder, inflammatory bowel disease (IBD), and antineoplastic treatments.

Some of the included studies reported the association between PhA and nutritional status through ROC curve analysis to generate cutoff points. Others compared stratified mean PhA values and the presence or absence of malnutrition. Some of the studies also presented correlation analyses between PhA and anthropometric indicators. Only one study evaluated concordance through the kappa value between PhA and the diagnosis of malnutrition. Some included studies reported more than one of these forms of association between PhA and nutritional status (Table 2). The associations are described below.

**Table 2:** Summary of evidence.

**References; location**	**Study design**	**Clinical health condition, sample size, sex, and age**	**Nutritional status indicator and reference for malnutrition**	**Principal results**	**Malnutrition prevalence**
**Studies with PhA cutoff analysis and nutritional status**					
Farias et al. (21); Brazil	Prospective study	**Hematopoietic stem cell transplantation** *n =* 67 58% M, 41% F 10.2 ± 4.1 years **Healthy children** *n =* 35 5–18 years	Malnutrition BMIz <−2 SD, WAZ <-1 SD Ideal weight <90% TSF <90%, AMC <90% WHO (22) Frisancho (23)	**ROC curve analysis for malnutrition** Cutoff, SPhA ≤0 SD Sensitivity of 92% and specificity of 70% AUC = 0.637 compared with WAZ **HSCT:** SPhA = 0.61 ± 0.98 **Healthy children:** SPhA = 1 ± 0.6 *p =* 0.054 **Pearson correlation coefficient with SPhA** BMIz: *r =* 0.457, *p <* 0.001 TSF: *r =* 0.370, *p <* 0.002 FFM: *r =* 0.375, *p <* 0.002 AMC: *r =* 0.412, *p <* 0.001 **Agreement diagnosis malnutrition with SPhA (kappa value)** BMIz <−2 SD, k= 0.026 (95% CI: −0.110 to 0.236) WAZ <−1 SD, k = 0.231 (95% CI: −0.240 to 0.493) Ideal weight <90%, k = 0.406 (95% CI: 0.197 to 0.611) TSF <90%, k = 0.435 (95% CI: 0.192 to 0.653) AMC <90% = 0.441 (95% CI: 0.190 to 0.672)	Not reported
Apostolou et al. (24); Greece	Cross-sectional study	**Chronic kidney disease** *n =* 30 66.6% M, 33.4% F 1–16 years	Weight z-score <−2 SD Height z-score <−1.88 SD BMIz <−2 SD AMAz <1.6 SD WHO (22) KDOQI guidelines (25)	**PhA cutoff point for malnutrition-stunting:** <3rd percentile **Pearson correlation coefficient with PhA** Weight: *r =* 0.483, *p <* 0.05 MUAC: *r =* 0.778, *p <* 0.001	**Malnutrition-stunting** Weight z-score 27% Height z-score 30% BMIz 20% AMAz 20% PhA <3rd percentile 30%
Popiolek et al. (20); Poland	Longitudinal study	**Anorexia nervosa** *n =* 46 100% F 16 ± 4.99 years	BMI Underweight: 16–18.5 kg/m^2^ Severely underweight: 15–15.99 kg/m^2^ Very severely underweight: <15 kg/m^2^ Starvation state: <16 kg/m^2^ WHO (26)	**ROC curve analysis for starvation** Cutoff: PhA > 4.93° Sensitivity of 38.96% and specificity of 100% AUC = 0.69 (95% CI: 0.53–0.82), *p =* 0.0164 compared with BMI (starvation state <16 kg/m^2^) **Entire population:** PhA = 4.27°± 1.18°**Starvation state:** *p =* 0.0299 BMI <16 kg/m^2^: PhA = 4.17° BMI ≥16 kg/m^2^: PhA = 4.52°**Correlation coefficient with PhA** Biceps muscle skinfold: rho = 0.341, *p =* 0.0204 AC: rho = 0.42, *p =* 0.0037 WHR: rho = 0.366, *p =* 0.0221	NA
Ashton et al. (27); United Kingdom	Prospective study	**Inflammatory bowel disease** *n =* 97 41.2% F, 58.8% M 14.49 years	BMIz Mild undernutrition: <-1 SD Moderate malnutrition: ≤-2 SD Overweight/obesity: > 1 and > 2 SD WHO Anthro software version 3.3.3 (2011)	**ROC curve analysis for BMIz** **>** **1 SD (overweight)** AUC = 0.460 (95% CI: 0.339–0.581), *p =* 0.487 **ROC curve analysis for BMIz** **<** **−1 SD (underweight)** AUC = 0.339 (95% CI: 0.265–0.532), *p =* 0.177 **Pearson correlation coefficient with PhA** BMIz: r^2^ = 0.02, *p =* 0.78	**BMIz** Moderate malnutrition 5.3% Mild undernutrition 8.5% Overweight/obesity 7.5%
**Studies comparing mean PhA values and the nutritional status**					
Bonaccorsi et al. (28); Italy	Cross-sectional study	**Eight-year-old children** *n =* 449 47% F, 53% M 8 years	BMI Not overweight: <19.4 kg/m^2^ Overweight: 19.4–25.6 kg/m^2^ Obesity: > 25.6 kg/m^2^ Cacciari et al. patterns (29)	**Male:** *p =* ns Not overweight: PhA = 6.4°± 0.6° Overweight/obesity: PhA = 6.4°± 0.6°**Female:** *p <* 0.05 Not overweight: PhA = 6.3°± 0.6° Overweight/obesity: PhA = 6.6°± 0.3°**Pearson correlation coefficient with PhA** (Male) BMI: *r =* 0.084, *p =* ns (Female) BMI: *r =* 0.336, *p <* 0.05	**Male** Overweight 21.3% Obesity 2.1% **Female** Overweight 13.9% Obesity 2.4%
Barufaldi et al. (30); Brazil	Cross-sectional study	**Indigenous children and adolescents** *n =* 3,204 50.6% M, 49.4% F 10.8 ± 2.9 years	Overweight BMIz > +2 z-score Stunting HAZ <2 z-score WHO (31)	**Entire population:** *p <* 0.001 Children: PhA=5.5°± 0.6° Adolescents: PhA= 6.1°± 0.8°**Children:** *p =* 0.004 Overweight: PhA = 5.7°± 0.5° Not overweight: PhA = 5.5°± 0.6°**Adolescents:** *p =* 0.006 Overweight: PhA = 6.3°± 0.7° Not overweight: PhA = 6.1°± 0.8°	**Children Overweight:** M = 5.5%, F = 5.9% **Stunting:** M = 16.2%, F = 15.0% **Adolescents Overweight:** M = 5%, F = 8.6% **Stunting:** M = 21.2%, F = 18.5%
Girma et al. (32); Ethiopia	Cross-sectional study	**SAM** *n =* 55 60% M, 40% F 36 ± 24 months **Healthy reference children** *n =* 80 47.5% M, 52.5% F 28 ± 15 months	**SAM** MUAC <11 cm or WFH <70% NCHS growth reference median and/or nutritional edema **Healthy children** WHZ and HAZ within ± 2 SD WHO (not specified)	**Healthy children:** PhA = 3.8°± 0.7°**SAM:** PhA = 2.2°± 0.7°*p <* 0.001 **SAM:** *p =* 0.12 Non-edematous: PhA = 2.4°± 0.8° Edematous: PhA = 2.1°± 0.6°*p =* 0.12 **Pearson correlation coefficient with PhA** MUAC: *r =* 0.31, *p <* 0.05 HAZ: *r =*−0.25, *p =* ns WAZ: *r =*−0.03, *p =* ns WFH: *r =* 0.19, *p =* ns	NA
Marino et al. (33); United Kingdom	Prospective study	**Primary ciliary dyskinesia** *n =* 43 51% M, 49% F 7.0 ± 5.2 years	Moderate malnutrition <−2 z-score of HAZ, WHZ, BMIz, and FFMIz WHO (22)	**Entire population:** PhA = 4.5°± 0.9°**Moderate malnutrition:** *p =* 0.0002 FFMIz <−2 z-score: PhA = 4.3°± 0.4° FFMIz >-2 z-score: PhA = 4.9°± 0.8°	**Moderate malnutrition** HAZ 4.6% BMIz 6.9% FFMIz 21%
Bourdon et al. (34); Blantyre, Malawi	Prospective observational study	**SAM** *n =* 183 54% M, 46% F 23 ± 12 months **Community participants** *n =* 42 62% M, 38% F 20.1 ± 12.3 months	WHZ, WAZ, or HAZ WHO (35)	**Community participants:** PhA = 3.8°± 0.8°**Edematous SAM:** PhA = 2.3°± 1.4°**Severe wasting:** PhA = 2.9°± 1.0° (*p <* 0.001)	Severe wasting 45.9% Edematous SAM 54%
Girma et al. (36); Ethiopia	Cross-sectional study	**SAM non-edematous** *n =* 136 56% M, 44% F Median 29 (IQR:14–60) months **SAM edematous** *n =* 214 57% M, 43% F Median 36 (IQR:24–60) months **Healthy children** *n =* 120 50% M, 50% F Median 38 (IQR: 22–82) months	**SAM** MUAC <11 cm or WFH <70% of the median of the NCHS growth reference and/or nutritional edema **Healthy children** WFH or BMI and HAZ within ± 2 SD of WHO (37)	**Healthy Children:** PhA = 4.3°± 1°**SAM:** PhA = 2.5°± 1.1°**SAM** Non-edematous: PhA = 2.8°± 1.2° edematous: PhA = 2.3°± 1°	NA
Macena et al. (38); Brazil	Cross-sectional study	**Children** **<** **5 years of age at risk or in chronic malnutrition** *n =* 100 46% M, 54% F 3 ± 0.78 years	WAZ, HAZ, BMIz, and MUAC/A WHO (31) HAZ Adequate: (z-score > −1) Risk of stunting (z-score ≤−1 and > −2) Stunted (z-score ≤2)	**Entire population:** PhA = 4.4°± 0.6°**HAZ:** *p =* 0.19 Adequate HAZ: PhA = 4.2°± 0.7° At-risk HAZ: PhA = 4.5°± 0.7° Stunted HAZ: PhA = 4.4°± 0.6°	**HAZ** Stunted 37% Risk of stunting 38%
**Studies that correlated PhA with the nutritional status**					
Nagano et al. (39 ); Japan	Cross-sectional study	**Malnourished patients** *n =* 10 100% M 2.6 ± 2.6 years **Well-nourished patients** *n =* 71 60.5% M, 39.5% F 3.36 ± 3.12 years	**Nutritional disturbance %IBW** Mild: 80%−90% Moderate: 70%−80% Severe: <70% Fukuoka reference **Nutritional disturbance %AMC** Mild: 80%−90% Moderate: 60–80% Severe: <60% Frisancho (40)	**Pearson correlation coefficient with PhA** IBW: *r =* 0.818, *p <* 0.001 AMC: *r =* 0.90, *p <* 0.001	**Nutritional disturbance %IBW** Mild: 70% Moderate: 30% **Nutritional disturbance %AMC** Severe: 10% Moderate: 20% Mild: 70%
Castro et al. (41); Brazil	Cross-sectional study	**Autism spectrum disorder** *n =* 63 81% M, 19% F 10.5 ± 4.1 years	BMI percentiles Underweight: ≥ 5th Healthy: > 5th to <85th Overweight: ≥ 85th to <95th Obesity: ≥ 95th CDC (42)	**Spearman correlation with PhA** BMI: *r =* −0.072, *p =* 0.05	Underweight 15.8% Overweight 38.9% Obesity 36.5%
Marino et al. (43); United Kingdom	Prospective study	**Congenital heart disease** *n =* 117 61% M, 39% F 44.3 ± 56 months	Moderate malnutrition HAZ and WAZ: <−2 z-score WHO (22)	**Entire population:** PhA = 4.2°± 1.3°**Pearson correlation coefficient with PhA** HAZ: *r =* 0.2, *p =* 0.03 WAZ: *r =* 0.3, *p =* 0.03	**Moderate malnutrition** Infants: HAZ = 28.5% Children: HAZ = 20.6%
Guimarães et al. (44); Brazil	Cross-sectional study	**Children receiving antineoplastic treatments**. *n =* 13 61.5% M, 38.5% F 103.2 ± 39.7 months	WAZ (0–10 years), WHZ (0–5 years), HAZ (0–19 years), BMIz (0–19 years) WHO reference. AM, AMC, and TSF Frisancho (45)	**Pearson correlation coefficient with PhA** Current weight: *r =* 0.920, *p <* 0.0001 AMC: *r =* 0.569, *p =* 0.042 AM: *r =* 0.618, *p =* 0.024 TSF: *r =* 0.471, *p =* ns	**BMIz** Risk of overweight 7.7% Overweight 23.2% Obesity 15.4% ^*^All the patients had correct height-for-age

AM, arm circumference; AMAz, arm muscle area z-score; AMC, arm muscle circumference; AN, anorexia nervosa; AUC, area under the curve; BIVA, bioelectrical vector analysis; BMIz, body mass index z-score; BW, body weight; CDC, Centers for Disease Control and Prevention; CHD, congenital heart disease; F, female; FFMIz, fat-free mass index z-score; HAZ, height-for-age z-score; HSCT, hematopoietic stem cell transplantation; IBW, ideal body weight; IQR, interquartile range; KDOQI, Kidney Disease Outcome Quality Initiative; M, male; MUAC, mid-upper arm circumference; MUAC/A, mid-upper arm circumference for age; NA, not applicable; NCHS, National Center for Health; ns, no significance; PhA, phase angle; SAM, severe acute malnutrition; SPhA, standardized phase angle; TSF, triceps skinfold thickness; WAZ, weight-for-age z-score; WFH, weight-for-height percentile; WHO, World Health Organization; WHR, waist-to-hip ratio; WHZ, weight-for-height z-score.

### Cutoff points for PhA

Among the 15 articles we included, only four reported potential cutoff points for PhA to detect patients with malnutrition. However, the clinical health conditions differed among the studies. Farias et al. (21) evaluated PhA as a standardized phase angle (SPhA), justifying that the use of this standardized variable could serve to compare among independent studies. After calculating PhA in grades (°), they standardized the data by using reference values for sex and BMI of the German population (46). The equation was: SPhA = {(Observed PhA [°]—PhA median for sex and BMI [°])/(standard deviation [SD] of the PhA for sex and BMI)}. They found the SPhA cutoff point of ≤0 SD to detect malnutrition in patients who had received hematopoietic stem cell transplantation (10.2 ± 4.1 years old), and the nutritional indicator reference was weight-for-age z-score (WAZ). This cutoff point had 92% sensitivity and 70% specificity, with an AUC of 0.637. The mean ± SD SPhA for patients who had received hematopoietic stem cell transplantation was 0.61 ± 0.98. The authors also evaluated the agreement between nutritional indicators and SPhA (≤0 SD) for the malnutrition diagnosis. They reported a kappa value of 0.026 (95% CI: −0.110 to 0.236) for body mass index z-score (BMIz) (<-2 SD), a kappa value of 0.231 (95% CI: −0.240 to 0.493) for WAZ (<-1 SD), and a kappa value of 0.406 (95% CI: 0.197 to 0.611) for ideal body weight (IDW) <90%. They reported the greatest agreement for TSF (<90%) (κ = 0.435 [95% CI: 0.192 to 0.653]) and arm muscle circumference (AMC) (<90%) (κ = 0.441 [95% CI: 0.190 to 0.672]).

Apostolou et al. (24) evaluated PhA as percentiles. They measured 400 children aged 2–18 years with BIA and classified the PhA values as percentiles derived from studies in the national pediatric population. They reported a PhA cutoff point for malnutrition-stunting of <3rd percentile for children with CKD (1–16 years old), but they did not report the sensitivity and specificity. Based on this PhA cutoff, they reported a prevalence of 30% malnutrition-stunting. Popiolek et al. (20) evaluated PhA as a crude variable in grades (°). They estimated a PhA cutoff of > 4.93° to identify the starvation state defined by BMI <16 kg/m^2^ in patients with anorexia nervosa (17.38 ± 4.99 years old), with 38.96% sensitivity and 100% specificity. The ROC curve built for BMI had an AUC of 0.69 (95% confidence interval [CI] 0.53–0.82, *p* = 0.0164). The mean ± SD PhA for the entire population was 4.27° ± 1.18°. They also reported the difference in PhA between female subjects who were in a state of starvation and those who were not (BMI <16 kg/m^2^, PhA = 4.17° vs. BMI ≥16 kg/m^2^, PhA = 4.52°, *p* = 0.0299). Ashton et al. (27) did not identify a clinically useful PhA cutoff associated with underweight (BMIz <−1) or overweight (BMIz > 1) in pediatric patients with IBD (mean 14.49 years old). The ROC curve analysis yielded an AUC of 0.460 (95% CI 0.339–0.581, *p* = 0.487) for overweight, and an AUC of 0.339 (95% CI 0.265–0.532, *p* = 0.177) for underweight.

### Studies that compared mean PhA values among nutritional status strata

**Figure 2:**
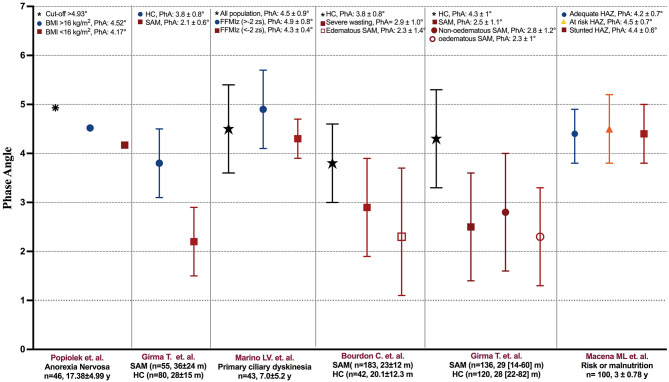
Phase angle reported in the studies stratified by the presence of undernourishment. PhA, phase angle; BMI, body mass index; FFMIz, fat-free mass index z-score; HAZ, height-for-age z-score; HC, healthy children; m, months; SAM, severe acute malnutrition; y, years.

Some authors compared the mean PhA among any conditions related to nutritional status (Figure 2). Bonaccorsi et al. (28) reported the PhA among 8-year-old children with excess BW or without being overweight. They used the following BMI criteria to diagnose excess BW: not overweight, <19.4 kg/m^2^; overweight, 19.4–25.6 kg/m^2^; and obesity, >25.6 kg/m^2^. There was no difference between boys with excess weight and without overweight (not overweight, PhA = 6.4° ± 0.6° vs. overweight/obesity, PhA = 6.4° ± 0.6°). However, in girls, there was a significant difference (not overweight, PhA = 6.3° ± 0.6° vs. overweight/obesity, PhA= 6.6° ± 0.3°, *p* < 0.05). Barufaldi et al. (30) reported the mean PhA values of indigenous children and adolescents (10.8 ± 2.9 years old); it was 5.5° ± 0.6° for children and 6.1° ± 0.8° for adolescents. They compared the mean PhA values between the strata of children and adolescents based on whether they had or did not have overweight based on BMIz. They defined overweight as BMIz > 2 z-score. There were differences in the mean PhA values of children with overweight compared with children without overweight (5.7° ± 0.5° vs. PhA= 5.5° ± 0.6°, *p* = 0.004). In addition, there was a significant difference in the mean PhA values between adolescents with and without overweight (6.3° ± 0.7° vs. 6.1° ± 0.8°, *p* = 0.006).

Girma et al. (32) evaluated the relationship between PhA and nutritional indicators in children (36 ± 24 months) with severe acute malnutrition (SAM), defined as MUAC <11 cm or weight-for-height percentile (WFH) <70%. The mean ± SD PhA of the group with SAM was different compared with healthy patients (28 ± 15 months) (2.2 ± 0.7° vs. 3.8 ± 0.7°, *p* < 0.001). Additionally, they compared the SAM group considering the presence of edema: PhA was not different between the non-edematous and edematous groups (2.4° ± 0.8° vs. 2.1° ± 0.6°, *p* = 0.12).

Marino et al. (33) reported a mean ± SD PhA of 4.5° ± 0.9° in children (7.0 ± 5.2 years old) with primary ciliary dyskinesia. In the study, they evaluated moderate malnutrition with the indicators height-for-age z-score (HAZ), weight-for-height z-score (WHZ), BMIz, and fat-free mass index z-score (FFMIz). They also compared PhA with FFMIz. Patients with moderate malnutrition (FFMIz <-2 z-score) has significantly lower PhA than patients who did not present moderate malnutrition (FFMIz >−2 z-score) (4.3° ± 0.4° vs. 4.9° ± 0.8°, *p* = 0.0002). Bourdon et al. (34) also reported the PhA of community participants and children with SAM (20.1 ± 12.3 months), whom they divided into the severe wasting group and the edematous group. They evaluated SAM with the indicators WAZ or HAZ based on the World Health Organization (WHO) definitions. Children with edematous SAM had lower PhA compared with community participants (2.3° ± 1.4° vs. 3.8° ± 0.8°, *p* < 0.001). In addition, children with severe wasting had lower PhA compared with community participants (2.9° ± 1.0° vs. 3.8° ± 0.8°, *p* < 0.001).

Girma et al. (36) evaluated PhA and nutritional indicators in children with non-edematous SAM (median 29 [IQR:14–60] months) and children with edematous SAM (median 36 [IQR: 24–60] months), who were defined as MUAC <11 cm or WFH <70% of the median of the National Center for Health (NCHS) growth reference and/or nutritional edema. They also reported PhA in healthy children (HC) (median 38 [IQR: 22–82] months). The mean ± SD PhA was 4.3° ± 1.0° in HC and 2.8° ± 1.1° in children with SAM. When stratifying the SAM group, the mean ± SD PhA was 2.8° ± 1.2° for the non-edematous group and 2.3° ± 1.0° for the edematous group. Macena et al. (38) evaluated children at risk or in chronic malnutrition (3 ± 0.78 years old) based on HAZ and stratified as: adequate (HAZ >-1), risk of stunting (HAZ <−1 and >-2), or stunted = (HAZ <2). The overall mean ± SD PhA was 4.4° ± 0.6°, and there was no significant difference between the adequate HAZ group (PhA = 4.2° ± 0.7°) and the at-risk HAZ (PhA = 4.5° ± 0.7°) and stunted HAZ (PhA° = 4.4 ± 0.6°) groups (*p* = 0.19).

### Studies that reported correlations between PhA and the nutritional status

Most of the studies correlated the nutritional indicators with PhA. In this sense, we considered the classification of cutoff points of correlations as very strong (r > 0.8), moderately strong (*r* = 0.6–0.8), fair (*r* = 0.3–0.6), and poor (r <0.3) associations (47). Nagano et al. (39) showed very strong positive correlations between PhA and IBW (*r* = 0.818, *p* < 0.001) and AMC (*r* = 0.90, *p* < 0.001) in malnourished patients. Guimarães et al. (44) reported in children receiving antineoplastic treatments a positive very strong correlation between PhA and current weight (*r* = 0.920, *p* < 0.0001), and a positive moderately strong correlation between PhA and arm circumference (AC) (*r* = 0.618, *p* = 0.024). They also reported a fair correlation between PhA and two anthropometric variables: TSF (*r* = 0.471 p > 0.05) and AMC (*r* = 0.569, *p* = 0.042).

Apostolou et al. (24) reported a positive moderately strong correlation between PhA and MUAC (*r* = 0.778, *p* < 0.001) and a positive fair correlation between PhA and BW (*r* = 0.483, *p* < 0.05) in patients with CKD. Farias et al. (21) found a fair correlation between PhA and BMIz (*r* = 0.457, *p* < 0.001), TSF (*r* = 0.370, *p* < 0.002), FFM (*r* = 0.375, *p* < 0.002), and AMC (*r* = 0.412, *p* < 0.001) in patients who had received hematopoietic stem cell transplantation. Popiolek et al. (20) found a fair correlation between PhA and biceps muscle skinfold (rho = 0.341, *p* = 0.0204), AC (rho = 0.42, *p* = 0.0037), and the waist-to-hip ratio (rho = 0.366, *p* = 0.0221) in adolescents with anorexia nervosa.

Bonaccorsi et al. (28) stratified 8-year-old children by sex and analyzed the correlation between PhA and BMI. Only girls showed a positive fair correlation (*r* = 0.336, *p* < 0.05). Marino et al. (43) studied PhA in children with CHD (44.3 ± 56 months) and reported an average PhA of 4.2° ± 1.3°. They reported a fair correlation between PhA and WAZ (*r* = 0.3, *p* = 0.03) and a poor correlation between PhA and HAZ (*r* = 0.2, *p* = 0.03). Girma et al. (32) only found a positive fair correlation between PhA and MUAC (*r* = 0.31, *p* < 0.05) in children with SAM, without correlations with HAZ, WAZ, and WHZ. Castro et al. (41) and Ashton et al. (27) found no significant associations between PhA and BMI in children with autism spectrum disorder (10.5 ± 4.1 years old) and in pediatric patients with IBD (14.49 years old), respectively.

### Prevalence of malnutrition based on any nutritional indicator

**Figure 3:**
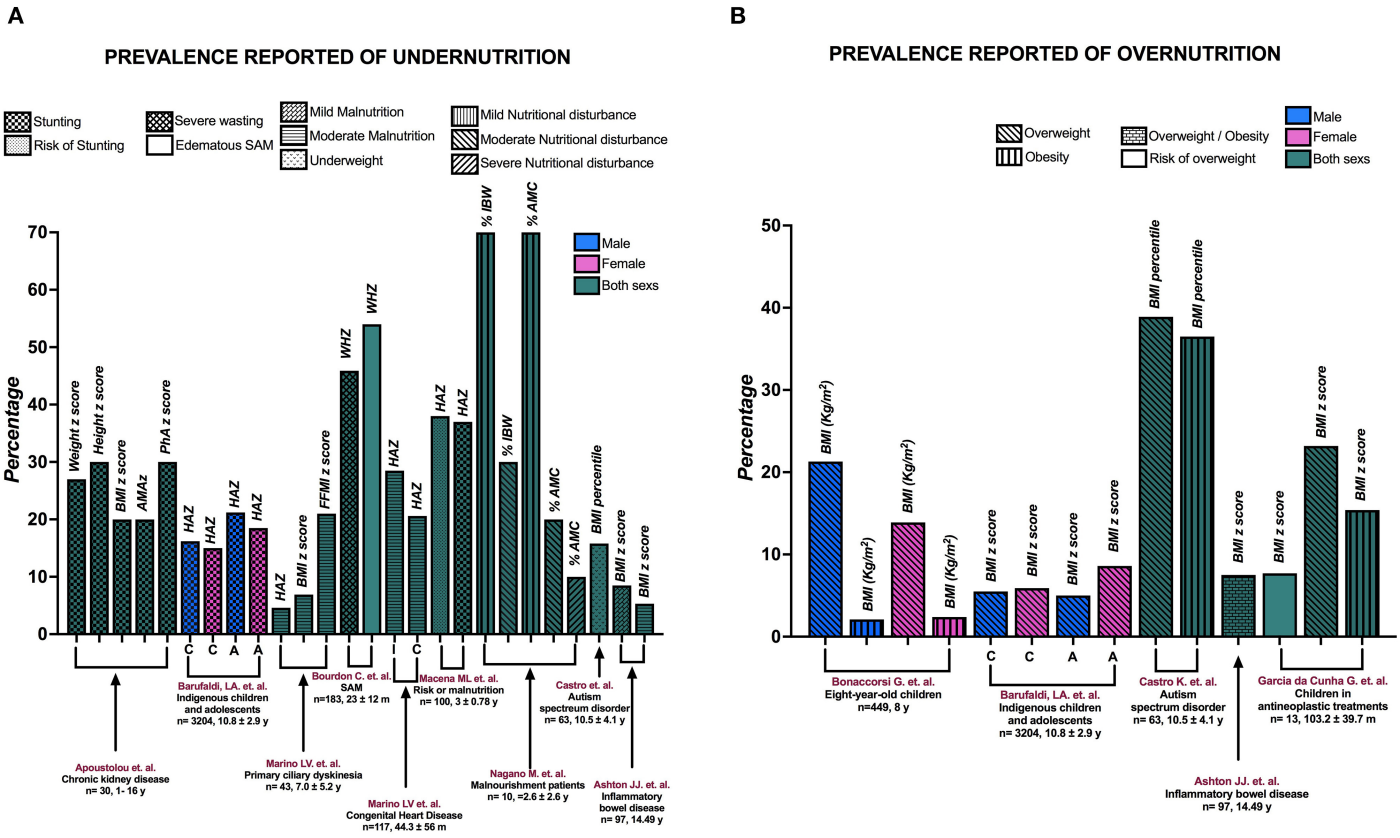
The prevalence of **(A)** undernutrition and **(B)** overnutrition reported in the studies. A, adolescents; AMAz, arm muscle area z-score; BMI, body mass index; C, children; FFMI, fat-free mass index; HAZ, height-for-age z-score; I, infants; SAM, severe acute malnutrition; WHZ, weight-for-height z-score; y, years; %AMC, percentage of arm muscle circumference; %IBW, percentage of ideal body weight.

The reported prevalence of malnutrition varies among pediatric populations. There is no unified way to carry out this classification due to variations in anthropometric indicators as well as the type of pathology. Thus, as a secondary objective, we have reported the prevalence of malnutrition and the anthropometric indicators that have been used to evaluate it (Figure 3).

Apostolou et al. (24) reported the prevalence of malnutrition-stunting based on various indicators: It was 27% based on weight z-score, 30% based on height z-score, 20% based on BMIz, and 20% based on arm muscle area z-score (AMAz). They also reported a 30% prevalence of malnutrition-stunting in children based on the PhA cutoff point (<3rd percentile). On the other hand, Marino et al. (33) reported a prevalence of moderate malnutrition with HAZ in 43 children with primary ciliary dyskinesia, with a prevalence of 28.5% in infants and 20.6% in children. In another study from the same group (43), the authors considered 117 patients with CHD and reported the prevalence of moderate malnutrition with different indicators: 4.6% based on HAZ, 6.9% based on BMIz, and 21% based on FFMIz.

In children with SAM, Bourdon et al. (34) found that 45.9% had severe wasting and 54% had edematous SAM. Macena et al. (38) reported that in a population with risk of malnutrition, 37% had stunting and 38% had a risk of stunting. Nagano et al. (39) included malnourished patients and reported that according to the percentage of ideal body weight (%IBW), 70% of the children had mild malnutrition and 30% had moderate malnutrition; according to the percentage of arm muscle circumference (%AMC), 10% had severe malnutrition, 20% had moderate malnutrition, and 70% had mild malnutrition. Two studies included extreme malnutrition. Barufaldi et al. (30) reported the prevalence of stunting and overweight in indigenous children and adolescents stratified by sex. They reported that 16.2% of boys and 15.0% of girls had stunting, and 21.2% of male adolescents and 18.5% of female adolescents had stunting. In addition, the prevalence of overweight in the population was 5.5% for male children, 5.9% for female children, 5% for male adolescents, and 8.6% for female adolescents. Castro et al. (41) included people with autism spectrum disorder and reported a prevalence of 15.8% for underweight, 38.9% for overweight, and 36.5% for obesity. Finally, Ashton et al. (27) found a prevalence of 5.3% for moderate malnutrition, 8.5% for mild malnutrition, and 7.5% for overweight/obesity in children with IBD.

Two studies only reported the prevalence of excess BW. Bonaccorsi et al. (28) included 449 8-year-old children and registered 21.3% of male children with overweight, 2.1% of male children with obesity, 13.9% of female children with overweight, and 2.4% of female children with obesity. Guimarães et al. (44) observed a prevalence of 7.7% for the risk of being overweight, 23.2% for overweight, and 15.4% for obesity in children receiving antineoplastic treatments.

### Bioimpedance model and usage specifications of the studies

According to the type of BIA, the researchers used different models and reported the usage specifications in the methodology (Supplementary Table 1). The approaches were diverse according to the type of device used and the type of pathology in which they made the electrical impedance measurements. Among the variations used, BIA included monofrequency (50 kHz) (34) and others used several frequencies (5, 50, 100, and 200 kHz) (36). Within more specific descriptions for the type of pathology, such as the study carried out in patients with CKD (24), patients were measured 1 h after dialysis so that the body fluid compartments were as close to healthy levels as possible. Patients who had received hematopoietic stem cell transplantation were evaluated in the absence of intravenous hydration and fever (21).

An important issue in the estimation of the PhA is the position in which the measurement is made because there could be differences in the BIA results between positions. Among the included studies, nine reported results from the supine position, and three reported results from the lying position, the horizontal position, and the lying in the supine position; however, in three of them, the position of measurement was not reported (24, 33, 44). Of the 15 studies we included, six studies reported the formula they used to calculate PhA (21, 28, 30, 36, 38, 39). Four of the studies did not report the formula (20, 27, 33, 43), but they used the same bioimpedance model reported by Małecka-Massalska et al. (48), who presented the formula in a Supplementary Table. Three other studies did not report the formula, but it was the same model reported by Girma et al. (36), who did report the formula in their study. Finally, for the two studies that did not report the formula, we took the information provided by the manufacturer (Supplementary Table 1).

### Risk of bias

The quality rating was acceptable, with a moderate risk of bias, in 11 of the studies assessed with the Quality Assessment Tool for Observational Cohort and Cross-Sectional Studies. Of these 11 studies, four studies were rated as fair, with some susceptibility to risk of bias, and ssevven studies had an overall good quality rating (Supplementary Table 2). The four studies assessed with the Quality Assessment of Diagnostic Accuracy Studies showed a low risk of bias in almost all domains (Supplementary Table 3).

## Discussion

In this present systematic review, we have synthesized the evidence derived from 15 studies aimed at evaluating the association between PhA and the nutritional status in pediatric populations. Four studies reported different PhA cutoff points. However, it is difficult to compare these cutoff points because the authors reported PhA differently: SPhA (21), percentiles (24), and degrees (20). Ideally, PhA could be standardized so that comparisons could be made among different populations. An alternative would be to report SPhA. However, this measure has a limitation because it is based on reference values of the evaluated population. Although Farias et al. (21) standardized PhA with reference values from the German population (46), not all populations have reference values, and it would be difficult to standardize the measurement.

Another variable that plays an important role is the anthropometric indicator, which is used as a reference standard. Among the studies that reported a cutoff point from ROC curve analysis, the anthropometric indicators were diverse. For example, some authors considered WAZ associated with malnutrition, while another study used BMI to detect the state of starvation defined as BMI <16 kg/m^2^ or BMIz associated with overweight. When using different anthropometric indicators as a reference standard, the state of nutrition is variable due to the lack of uniform definitions by heterogeneous nutrition screening practices (2). Although a uniform definition of malnutrition in children is not available, the indicator or construct that best defines malnutrition should be standardized in this population, in such a way that it is used as a reference standard, similar to what is found in adult populations with the Global Leadership Initiative on Malnutrition (GLIM) construct (49).

The predictive values of PhA associated with malnutrition evaluated with WAZ had a sensitivity of 92% and a specificity of 70% in patients who had received hematopoietic stem cell transplantation. These values are higher than the predictive values of nutritional screening tools such as STRONGkids (50). In a study carried out to validate this nutritional screening tool, the authors used alterations in one of these anthropometric indicators as a reference for malnutrition: WAZ, weight-for-height, and HAZ, resulting in a sensitivity of 86% and a specificity of 72% (51). For both PhA and the nutritional screening tools, there was greater sensitivity than specificity, which is important when it comes to creating a diagnostic tool, because more positive cases of malnutrition can be detected when used in the population, leading to early interventions.

In acute malnutrition, there is a displacement of intracellular fluids to the extracellular space, leading to a significant decrease in BCM and, consequently, a reduction in PhA (52). We observed this trend in studies comparing patients with SAM to HC, where the observed mean PhA in the SAM group was lower (2.1°-2.8°) compared with HC (3.8°-4.3°) (32, 34, 36). However, these values are different from that reported by Macena et al. (38), who observed no differences in PhA between the population with chronic malnutrition (HAZ <2) or risk of chronic malnutrition (HAZ <-1 and > −2) and those with adequate HAZ. This phenomenon can be explained by the fact that in chronic malnutrition, there could be an adaptive response to energy restriction that, when there are periods of energy availability, could favor fat storage to the detriment of its use (53). This fat accumulation would increase Xc and, therefore, increase PhA. These changes would explain why a smaller PhA is not observed in children with stunting.

We included studies that showed very strong correlations between PhA and current weight, AMC, and % IBW. These correlations could be explained by the fact that PhA is a parameter that reflects BCM (54): a decrease in BCM is associated with a decrease in Xc and a decrease in TBW, and PhA decreases alongside a reduction in Xc and an increase in R. BCM is the functional mass, that is, the total mass of all the cellular elements representing the metabolically active components of the body. It is calculated from raw impedance electrical data and height (55), and it is not affected by the hydration status (56). Finally, AMC reflects the total body protein store (57); it is an indicator of skeletal muscle mass, which comprises most of BCM. On the other hand, the fact that PhA in malnourished children (%IBW <90%) is less than that of well-nourished children indicates that the relationship between BCM and BW is lower in malnourished children than in HC (39). The available evidence suggests that nutritional management with high-calorie, high-protein oral nutritional supplementation with β-hydroxy-β-methylbutyrate increases BW, AMC, and PhA, with a decrease in R and an increase in Xc (56).

Some of the included studies reported associations between PhA and BMI. For example, Farias et al. (21) reported an association with *r* = 0.457 (*p* < 0.001), and Bonaccorsi et al. (28) reported an association with *r* = 0.336 (*p* < 0.05) but only in female patients. However, the evidence shows that in both adult and pediatric populations, BMI is associated with PhA independently of age and sex. The similar magnitude of the association is similar in pediatric subjects (*r* = 0.31, *p* < 0.001) but weak in adults (*r* = 0.03, *p* < 0.001) (46). Interestingly, only two articles reported correlations with anthropometric indicators in pediatric populations, and they showed different trends. Girma et al. (32) found no correlations between PhA and HAZ and WAZ in patients with SAM. However, Marino et al. (43) observed a positive correlation between PhA and HAZ (*r* = 0.2, *p* = 0.03) and WAZ (*r* = 0.3, *p* = 0.03) in patients with CHD. This discrepancy could be due to several factors, such as the population studied and small sample size. Thus, more evidence is needed to determine whether PhA and anthropometric indicators such as HAZ and WAZ really correlate with each other.

We include studies that reported PhA at 50 kHz because these data show good reproducibility. In the literature, this frequency has been used to determine and predict health in healthy populations, and this same frequency has been used to confirm alterations in populations with different diseases and also by examining intracellular and extracellular fluids (4, 58). At frequencies below 5 kHz and above 200 kHz, poor reproducibility has been noted, especially for the reactance at low frequencies. Moreover, most single-frequency BIA analyzers operate at 50 kHz (4) and 50 kHz has been used to estimate body composition (59).

It is important to highlight the development of different techniques used in nutritional evaluation, which can contribute to the comprehensive evaluation of patients. Nevertheless, there are some important considerations when comparing the results between populations and with subsequent measurements in the same patient. It is necessary to consider the position in which the impedance measurement is performed. Wiech et al. (60) demonstrated differences between measurements in the lying, sitting, and standing positions, and they analyzed their data by considering sex. In men, there was a significant difference in PhA when it was measured in the sitting position (7.23 ± 1.40) compared with the standing position (6.27 ± 0.68) (*p* = 0.020). On the other hand, in women, there were differences between the lying and standing positions (6.35 ± 1.55 vs. 5.40 ± 0.72, *p* = 0.022) and between the sitting and standing positions (6.56 ± 1.54 vs. 5.40 ± 0.72, *p* = 0.004). These position-related differences in impedance measurements are due to the fact that it impacts the resistance and reactivity measurements and is finally reflected in the intracellular and extracellular hydrated tissue and its cell mass. We recommend that researchers report the position of the measurements and make subsequent measurements in the same patients in the same position to avoid bias.

In adult populations with different diseases, PhA is a recognized nutritional status marker (16, 61–63). However, malnutrition is a complex multifactorial phenomenon, and several factors could be associated with inflammation, catabolism, and the presence of edema. These factors could also have an impact on impedance markers, such as PhA (52). In the field of pediatric nutrition, there is still a lack of evidence to fully support the current hypothesis that PhA is a prognostic factor for clinical outcomes and a nutritional status marker. The evidence presented supports an association between PhA and malnutrition in pediatric populations. However, specific PhA cutoff points cannot yet be presented, and additional research is needed.

The present review presents some limitations. The main limitation is the inability to compare the studies due to the variety of BIA models used to obtain PhA and the different ways the researchers reported PhA (SPhA, percentiles, and degrees). In addition, the researchers used several nutritional indicators as a reference for malnutrition, and the included studies focused on several different conditions. Hence, it is difficult to generalize the information reported in this review. However, a strength of this review is that we included studies that evaluated PhA cutoff points and the association between PhA and malnutrition, that showed an association between PhA and anthropometric indicators, and that compared the mean PhA values in the presence and absence of malnutrition. Furthermore, we considered different age groups and different disease entities. Although this approach introduced heterogeneity, we summarized a larger picture of PhA and its association with the nutritional status of pediatric patients.

Carrying out a critical evaluation of our systematic review, our results are considered valid because the chosen articles are pertinent and important to answer the research question. We tried to assess the quality of the studies, although it was difficult to compare the PhA cutoff points, as mentioned above. Ideally, studies should standardize PhA to allow for comparisons across different populations. Nevertheless, our review summarizes a broader picture of PhA and its association with the nutritional status of pediatric patients. Our review demonstrates the benefit of measuring PhA: It is an easy-to-obtain parameter and applicable to address the diagnosis of malnutrition.

## Conclusion

The early identification of malnutrition is relevant to establish the correct nutritional treatment and increase the positive clinical outcomes of inpatient children. This endeavor encourages the development of sensitive markers that can detect malnutrition early in the course of the disease. PhA is generally easy to measure, and it can be seen as a complementary parameter to diagnose malnutrition because it cannot evaluate the entire construct of the nutritional condition. Although the results of this systematic review are inadequate to establish PhA cutoff points associated with malnutrition in pediatric populations, additional research in various pathologies as well as a consensus on malnutrition construct in pediatric populations could be seen as an area of opportunity for future studies.

## Data availability statement

The original contributions presented in the study are included in the article/Supplementary material, further inquiries can be directed to the corresponding author.

## Author contributions

IM-V and AÁ-N designed the research. AF-O, AÁ-N, EAR-A, AT-M, ADG-G, BAP-N, JF-S, MG-C, and IM-V performed the research and drafted the manuscript. AF-O, AÁ-N, EAR-A, AT-M, JF-S, and IM-V analyzed the data. All authors contributed to the article and approved the submitted version.
