# Supplementary material for: Association between phase angle and the nutritional status in pediatric populations: a systematic review
Source: Front Nutr. 2023 Jun 21;10:1142545. doi: 10.3389/fnut.2023.1142545 (PMC10320581; doi:10.3389/fnut.2023.1142545)
Supplement: Supplementary file 1 [file Data_Sheet_1.docx]

Supplementary Material

Association of phase angle with nutritional status in Pediatric populations: A systematic review

**Franco-Oliva Andrea^1,^**^†^**, Ávila-Nava Azalia^2,^**^†^**, Rodríguez-Aguilar Estíbaliz Amairani^1^, Trujillo-Mercado Ander^3^, García-Guzmán Alda Daniela^3,4^, Pinzón-Navarro Beatriz Adriana^3,5^, Fuentes-Servín Jimena^1^, Guevara-Cruz Martha ^3,6^, Medina-Vera Isabel^1,3*^.**

^†^These authors contributed equally to this work and share first authorship.

*** Correspondence:** Isabel Medina-Vera: [isabelj.medinav@gmail.com](mailto:isabelj.medinav@gmail.com)

# Supplementary Data

# Supplementary Table 1. Bioimpedance model and usage specifications of the studies.

| **Author, year** | **Bioimpedance model** | **Bioimpedance usage specifications** | **PhA estimation formula used** | **Measurement position reported** |
| --- | --- | --- | --- | --- |
| Farias et al. 2013 | Tetrapolar model RJL Quantum | The patient in supine position, arms, and legs apart, in the absence of intravenous hydration and fever. | PhA: [Arc tangent (Xc/R)] x (180/ π). | Supine position |
| Apostolou A. et al. 2014 | Bodystat Quadscan 4000, Bodystat, (Beaconsfield, UK) according to BIA protocol | All continuous ambulatory peritoneal dialysis (CAPD) patients were measured one hour after dialysis so that body fluid compartments were as closer to healthy levels as possible. | * PhA: the angle the impedance vector forms relative to the R vector (atan (Xc/R) x 180/ π ).  * The formula found in another study where the same model was used is reported. (1) | Not reported |
| Popiolek et al. 2019 | ImpediMed bioimpedance analysis SFB7 BioImp v1.55 (Pinkenba, Brisbane, Qld 4008, Australia) | The measurements were taken in the horizontal position with limbs resting loosely at 30–45 degrees to the body. Before the examination, patients had to lie in position for 5 min and were not allowed to drink, eat, or make any physical effort in the preceding three hours. Measurements were taken in triplicate. The measurement was conducted at different frequencies: 50 kHz for PhA and Cm as the recognized standard, and 5 kHz and 200 kHz for Z. The Z200/5 parameter was defined as Z at 200 kHz, and a 5 kHz ratio was then calculated. | *PhA: arctangent ratio Xc: R and transformation of radians to degrees – previous result multiplied by 180˚/π  * The formula found in another study where the same model was used is reported. (2) | Horizontal position |
| Ashton et al. 2021 | Impedance iMedSFB7 (Pinkenba, QLD 4008 Australia) | The machine was calibrated before use with a circuit of known impedance, as per manufacturer's guidelines. Measurements were taken in triplicate, measurements were conducted in unfasted subjects using a standard tetrapolar electrodes distribution whilst the patients were supine. this is a single-channel tetra-polar device measuring resistance, reactance and phase angle across up to 256 frequencies. | *PhA: arc-tangent ratio Xc: R and transformation of radians to degrees – previous result multiplied by 180˚/π*  * The formula found in another study where the same model was used is reported. (2) | Supine position |
| Bonaccorsi et al. 2009 | STA/BIA Soft Tissue Analyzer (AKERN, Florence, Italy) | The analysis was conducted in vectorial and conventional ways. For vectorial interpretation, we used BIVA software 2002, BIVA Tolerance e Confidence Files. The conventional interpretation of BIA was done using Bodygram software (AKERN). All measurements were taken with the tetrapolar impedance method, based on the application of a constant, low- level (0.8 mA), alternating (50 kHz) current administered to the human body. Measurements were taken while the children, without shoes and socks, were lying supine on a cot. Two self-adhesive current-introducing electrodes were positioned in the middle of the dorsal surfaces of the right hand and foot proximal to the metacarpal–phalangeal and metatarsal–phalangeal joints, respectively. Two self-adhesive detector electrodes were placed medially between the distal prominences of the radius and the ulna and between the medial and lateral malleolus at the ankle. | PhA: arctangent of (X/R). | Lying on supine position |
| Barufaldi et al. 2011 | Quantum II® bio-electrical impedance analyzer (RJL Systems, Clinton Township, Michigan, United States). | The measurements were performed with the individual in the supine position on a coated mattress, free of electricity-conducting material, and at ambient temperature. Contact with the legs, arms, and trunk was avoided; the electrodes were placed according to the manufacturer’s instructions. In addition, subjects had refrained from any intense physical activity in the 4 hours prior to measurement. | PhA: arctangent (reactance/resistance) × 180°/π | Supine position |
| Girma et al. 2018 | Quadscan 4000 analyser (Bodystat, UK) | In brief, self-adhesive disposable electrodes were attached at the right hand and foot, injecting leads were connected to the electrodes just behind the finger and toe and the measuring leads were then connected to the electrodes on the right wrist and right ankle. Measurements were taken in triplicate 5 min apart, with children supine and limbs abducted. | PhA:(atan (Xc/R) x 180/π  * The formula found in another study where the same model was used is reported. (1) | Supine position |
| Marino et al. 2019 | Bioelectrical impedance Spectroscopy ImpediMedSFB7 (Pin-kenba, QLD 4008 Australia) | The machine was calibrated before use with a circuit of known impedance provided by the manufacturer. Measurements were completed in unfasted subjects. Measurements were conducted using a standard tetrapolar electrodes distribution; the inner arm electrode (sensor) was placed on the dorsal surface of the right wrist and the leg electrode was placed on the anterior surface of the right ankle. | *PhA:arc-tangent ratio Xc: R and transformation of radians to degrees – previous result multiplied by 180˚/π*  * The formula found in another study where the same model was used is reported. (2) | Not reported |
| Bourdon et al. 2021 | Bioelectrical Impedance Analyzer (Bodystat QuadScan4000) | As per manufacturer's instructions, four self-adhesive disposable electrodes provided by manufacturer were attached in a standard tetrapolar position. The two distal electrodes, one on the dorsal side of the hand next to the metacarpal-phalangeal joint and the other on the foot next to the metatarsal-phalangeal joint, were connected to injection leads. The two proximal electrodes were connected to measuring leads and positioned on the forearm and leg. A spacing of > 5.5 cm was maintained between the distant and proximal electrodes. This positioning has been recommended to prevent interactions between electrodes. To ensure a supine position with legs and arms extended, research staff guided mothers to verbally support their child and use distraction methods (e.g., watching cartoons at bedside). Care was taken that children were well positioned, untouched by mothers, and not in contact with conductive materials (e.g., bed frame). Measurements were taken in triplicates, but tests were repeated up to 5 times if the variance in R or Xc was above 5%. The coefficient of variation for PhA was evaluated when cleaning the database and if above 10% either the discrepant replicate was identified and removed, or the measurement was rejected as unreliable. Measurements at 50 kHz. | *PhA: the angle the impedance vector forms relative to the R vector (atan (Xc/R) x 180/ π ).  * The formula found in another study where the same model was used is reported. (1) | Supine position |
| Girma et al. 2021 | Quadscan 4000 analyser (Bodystat, UK) | Measurements were taken in triplicate, each spaced 5 min apart, while children were supine on a stretcher with limbs abducted from the body. Four frequencies (5, 50, 100 and 200 kHz) | PhA: the angle the impedance vector forms relative to the R vector (atan (Xc/R) x 180/ π ). | Supine position |
| Macena et al. 2021 | Tetrapolar BI1010 equipment (Sanny®, Sao Paulo, Brazil) | 3 h of fasting and emptying the bladder up to 15 min before the test of the procedure. The measurement took place with the participants lying supine, wearing light clothing, barefoot, and without metallic ornaments. Four electrodes were attached to the child's right hemibody: on the wrist, between the distal prominences of the radius and ulna; in the hand, close to the metacarpophalangeal joint on the dorsal surface; at the ankle, between the medial and lateral malleoli; and in the foot, in the transverse arch of the upper surface. Measurements at mono-frequency (50 kHz). | PhA: (Xc/R) x (180º/ π) | Supine position |
| Nagano et al. 2000 | RJL spectrum (RJL system, Detroit, MI). | A fixed-frequency current (50 KHz, 800 μA) was applied between the distal end of the right third metacarpal bone and the distal end of the right third metatarsal bone. Electrical Rz and Xc were obtained from the reduction in electrical potential between the extensor side of the right hand joint and the corresponding side of the right ankle joint. Stick-on type electrocardio-graphic electrodes were used. The contact areas were scrubbed with alcohol immediately before electrode placement and the electrodes were used only once. The measurements were made early in the morning before breakfast, with the patients kept in a supine position on the bed in such a way that the current would not pass through the bed during measurement. Those who were able to urinate were asked to do so before the measurements were begun. | PhA: atan (Xc/Rz) x 180/π | Supine position |
| Castro et al. 2017 | Biodynamics 450® version 5.1, Biodynamics. Corporation, Seattle, WA, USA | It was performed using Resting ECG tab electrodes (Conmed Corporation, Utica, NY, USA). Briefly, the measurements were done with the patients lying down with their legs and arms parallel to their bodies and positioned far from the chest. The electrodes were placed on standard locations (the dorsal surface of the right wrist, the third metacarpal bone, the anterior surface of the right ankle between the bone prominences, and the dorsal surface of the third metatarsal bone). Instructions were given to  the patients to be followed before BIA tests: overnight fasting, exercise restriction 24 hours before examination and bladder voiding. | **PhA: arctangent of (X/R)  **The formula found in Quick Start Guide for the BIA 450. (3) | Lying position |
| Marino et al. 2018 | ImpediMedSFB7 (Pin-kenba, QLD 4008 Australia) | Measurements were taken in unfasted subjects, in triplicate. Measurements were conducted using a standard tetrapolar electrodes distribution, on palms of hands and soles of feet, supine with arms and legs apart. Data files were processed using specialist software (Bioimp, ImpediMed), with data points rejected if they met any of the following criteria; i) positive X centre (Xc) values, ii) negative resistance values. PhA at a current frequency of 50 Hz was used for analysis. | PhA:arc-tangent ratio Xc:R and transformation of radians to degrees – previous result multiplied by 180˚/π*  * The formula found in another study where the same model was used is reported. (2) | Supine position |
| Guimarães et al. 2021 | Byodinamics®, model 450 brand (TB Brazil) | Body composition was evaluated according to the manufacturer’s recommendations. | ** PhA: arctangent of (X/R)  **The formula found in Quick Start Guide for the BIA 450. (3) | Not reported |

Information about PhA estimation formula was obtained by other sources* or **manufacture due to the study did not report it.

1. Girma T, Kaestel P, Molgaard C, Ritz C, Andersen GS, Michaelsen KF, et al. Utility of bio-electrical impedance vector analysis for monitoring treatment of severe acute malnutrition in children. Clin Nutr. 2021;40(2):624-31.

2. Malecka-Massalska T, Chara K, Smolen A, Kurylcio A, Polkowski W, Lupa-Zatwarnicka K. Bioimpedance vector pattern in women with breast cancer detected by bioelectric impedance vector analysis. Preliminary observations. Ann Agric Environ Med. 2012;19(4):697-700.

3. Quick Start Guide for the BIA 450. Chapter 8: Definitions. https://www.biodyncorp.com/pdf/quick_start_guide_450.pdf

**Table supplementary 2. Quality Assessment Tool for Observational Cohort and Cross-Sectional Studies.**

| **Criteria** | **Bonaccorsi et al. 2009** | **Barufaldi et al. 2011** | **Girma et al. 2018** | **Marino et al. 2019** | **Bourdon et al. 2021** | **Girma et al. 2021** | **Macena et al. 2021** | **Nagano et al. 2000** | **Castro et al. 2017** | **Marino et al. 2018** | **Guimarães et al. 2021** |
| --- | --- | --- | --- | --- | --- | --- | --- | --- | --- | --- | --- |
| 1. Was the research question or objective in this paper clearly stated? | Y | Y | Y | Y | Y | Y | Y | Y | Y | Y | Y |
| 2. Was the study population clearly specified and defined? | Y | N | Y | Y | Y | Y | N | N | Y | Y | Y |
| 3. Was the participation rate of eligible persons at least 50%? | Y | Y | Y | Y | Y | Y | Y | Y | Y | Y | Y |
| 4. Were all the subjects selected or recruited from the same or similar populations (including the same time period)? Were inclusion and exclusion criteria for being in the study prespecified and applied uniformly to all participants? | N | N | Y | N | Y | Y | N | N | Y | Y | Y |
| 5. Was a sample size justification, power description, or variance and effect estimates provided? | N | N | N | N | N | N | N | N | Y | N | N |
| 6. For the analyses in this paper, were the exposure(s) of interest measured prior to the outcome(s) being measured? | NA | NA | NA | NA | NA | NA | NA | NA | NA | NA | NA |
| 7. Was the timeframe sufficient so that one could reasonably expect to see an association between exposure and outcome if it existed? | NA | NA | NA | NA | NA | NA | NA | NA | NA | NA | NA |
| 8. For exposures that can vary in amount or level, did the study examine different levels of the exposure as related to the outcome (e.g., categories of exposure, or exposure measured as continuous variable)? | NA | NA | NA | NA | NA | NA | NA | NA | NA | NA | NA |
| 9. Were the exposure measures (independent variables) clearly defined, valid, reliable, and implemented consistently across all study participants? | Y | Y | Y | Y | Y | Y | Y | Y | Y | Y | Y |
| 10. Was the exposure(s) assessed more than once over time? | NA | NA | NA | NA | NA | NA | NA | NA | NA | NA | NA |
| 11. Were the outcome measures (dependent variables) clearly defined, valid, reliable, and implemented consistently across all study participants? | Y | Y | Y | Y | Y | Y | Y | Y | Y | Y | Y |
| 12. Were the outcome assessors blinded to the exposure status of participants? | NA | NA | NA | NA | NA | NA | NA | NA | NA | NA | NA |
| 13. Was loss to follow-up after baseline 20% or less? | NA | NA | NA | NA | NA | NA | NA | NA | NA | NA | NA |
| 14. Were key potential confounding variables measured and adjusted statistically for their impact on the relationship between exposure(s) and outcome(s)? | Y | Y | Y | N | Y | Y | N | N | Y | Y | Y |
| **Quality Rating (Good, Fair, or Poor)** | **Good** | **Fair** | **Good** | **Fair** | **Good** | **Good** | **Fair** | **Fair** | **Good** | **Good** | **Good** |
| **Comments (If POOR, please state why):** |  |  |  |  |  |  |  |  |  |  |  |

Y, Yes; N, No; NA, not applicable; NR, not reported. The Quality Rating of each study was rated as Good, Fair or Poor.

**Table supplementary 3.** Summary of risk of bias assessment and applicability (QUADAS-2 tool) of diagnostic accuracy studies.

| **Reference** | **Risk of bias** | | | | **Applicability** | | |
| --- | --- | --- | --- | --- | --- | --- | --- |
|  | **Patient selection** | **Index test** | **Reference Standard** | **Flow & time** | **Patient selection** | **Index test** | **Reference Standard** |
| **Farias et al. 2013** | **H** | **L** | **L** | **L** | **H** | **L** | **L** |
| **Apostolou et al. 2014** | **H** | **L** | **H** | **L** | **L** | **L** | **L** |
| **Popiolek et al. 2019** | **L** | **L** | **L** | **L** | **L** | **L** | **L** |
| **Ashton et al. 2021** | **H** | **L** | **L** | **L** | **L** | **L** | **L** |
| **L:** Low risk, **H:** High risk; **U:** Unclear. | | | | | | | |
